# Feasibility and Acceptability of an mHealth Delivered Mindfulness Therapy for Caregivers

**DOI:** 10.1093/geroni/igaa057.1010

**Published:** 2020-12-16

**Authors:** Elissa Kozlov, XinQi Dong

**Affiliations:** Rutgers University, New Brunswick, New Jersey, United States

## Abstract

Decades of research have documented the profound, negative effects of caregiving on unpaid caregivers. Mindfulness Therapy (MT) is a promising, non-pharmacological technique with proven efficacy and effectiveness in managing stress, depression and anxiety in diverse populations. While the evidence-base for MT in caregiving is growing, traditional MT (8+ hours of face-to-face treatment with trained providers) is likely not a realistic treatment model for most caregivers due to lack of trained personnel, time constraints of the caregiver, and reimbursement issues. Therefore, in order to meet the unique needs of caregivers of older adults with cognitive impairment, an innovative delivery model is required. MHealth can be a useful tool to deliver behavioral interventions, as it overcomes barriers of traditional psychotherapy such as provider availability, scheduling conflicts, and cost. The objective of this paper is to report the feasibility, acceptability and preliminary efficacy of a pilot trial of mHealth delivered MT for stress and caregiver burden in caregivers of persons with dementia. The average age of participant was 63.2 years old. After two weeks, 93% of participants reported using the mindfulness app for an average of 48.38 minutes per week. At eight weeks, 88% of users reported using the mindfulness app for an average of 35 minutes per week. At 8 weeks, 100% of users reported practicing mindfulness without using the app for an average of 45.6 minutes per week. MHealth mindfulness therapy appears to be a feasible method of delivering mindfulness to caregivers of older adults with memory impairment.

